# Preservation of tumour oxygen after hyperbaric oxygenation monitored by magnetic resonance imaging

**DOI:** 10.1054/bjoc.1999.0882

**Published:** 1999-12-08

**Authors:** Y Kinoshita, K Kohshi, N Kunugita, T Tosaki, A Yokota

**Affiliations:** Departments of NeurosurgeryHyperbaric MedicineEnvironmental Health, University of Occupational and Environmental Health, 1–1 Iseigaoka, Yahatanishi-ku, Kitakyushu 807-8555, Japan; Medical Business Group, Daido Hoxan Inc., Tokyo, Japan

**Keywords:** hyperbaric oxygenation, molecular oxygen, paramagnetism, relaxation time, magnetic resonance imaging

## Abstract

Hyperbaric oxygen (HBO) has been proposed to reduce tumour hypoxia by increasing the dissolved molecular oxygen in tissue. Using a non-invasive magnetic resonance imaging (MRI) technique, we monitored the changes in MRI signal intensity after HBO exposure because dissolved paramagnetic molecular oxygen itself shortens the T1 relation time. SCCVII tumour cells transplanted in mice were used. The molecular oxygen-enhanced MR images were acquired using an inversion recovery-preparation fast low angle shot (IR-FLASH) sequence sensitizing the paramagnetic effects of molecular oxygen using a 4.7 tesla MR system. MR signal of muscles decreased rapidly and returned to the control level within 40 min after decompression, whereas that of tumours decreased gradually and remained at a high level 60 min after HBO exposure. In contrast, the signal from the tumours in the normobaric oxygen group showed no significant change. Our data suggested that MR signal changes of tumours and muscles represent an alternation of extravascular oxygenation. The preserving tumour oxygen concentration after HBO exposure may be important regarding adjuvant therapy for cancer patients. © 2000 Cancer Research Campaign


*British Journal of Cancer* (2000) **82**(1), 88–92
© 2000 Cancer Research Campaign
Article no. bjoc.1999.0882

### Preservation of tumour oxygen after hyperbaric oxygenation monitored by magnetic resonance imaging

**Y Kinoshita[1], K Kohshi[1,2], N Kunugita[3], T Tosaki[4] and A Yokota[1]**

Departments of [1]Neurosurgery, [2]Hyperbaric Medicine and [3]Environmental Health, University of Occupational and Environmental Health, 1–1 Iseigaoka, Yahatanishi-ku, Kitakyushu 807-8555, Japan; [4]Medical Business Group, Daido Hoxan Inc., Tokyo, Japan

**Summary** Hyperbaric oxygen (HBO) has been proposed to reduce tumour hypoxia by increasing the dissolved molecular oxygen in tissue. Using a non-invasive magnetic resonance imaging (MRI) technique, we monitored the changes in MRI signal intensity after HBO exposure because dissolved paramagnetic molecular oxygen itself shortens the T1 relation time. SCCVII tumour cells transplanted in mice were used. The molecular oxygen-enhanced MR images were acquired using an inversion recovery-preparation fast low angle shot (IR-FLASH) sequence sensitizing the paramagnetic effects of molecular oxygen using a 4.7 tesla MR system. MR signal of muscles decreased rapidly and returned to the control level within 40 min after decompression, whereas that of tumours decreased gradually and remained at a high level 60 min after HBO exposure. In contrast, the signal from the tumours in the normobaric oxygen group showed no significant change. Our data suggested that MR signal changes of tumours and muscles represent an alternation of extravascular oxygenation. The preserving tumour oxygen concentration after HBO exposure may be important regarding adjuvant therapy for cancer patients. © 2000 Cancer Research Campaign

**Keywords:** hyperbaric oxygenation; molecular oxygen; paramagnetism; relaxation time; magnetic resonance imaging

Tumour oxygenation is known to enhance the efficacy of radiotherapy, because the presence of hypoxic tumour cells is considered to be one of the major reasons for failure to control tumours (Hall, 1994). Regarding hypoxic tumour cells, it is also known that ionizing radiation and some chemotherapeutic agents are less effective at low oxygen levels. Many studies on tumour oxygen tension levels using direct invasive measurements have been reported (Vaupel et al, 1984; Rampling et al, 1994; Brizel et al, 1996; Collingridge et al, 1997; Helmlinger et al, 1997; Al-Hallaq et al, 1998). Non-invasively, there has been increasing interest in measurements of changes in tissue oxygen tension using magnetic resonance imaging (MRI) methods. Semi-quantitative measurements of the tumour oxygen level have been discussed using oxygenation-sensitive $^{1}$H-MRI measurements during 100% oxygen inhalation (Karczmar et al, 1994; Kuperman et al, 1995; Edelman et al, 1996; Oikawa et al, 1997; Tadamura et al, 1997; Obata et al, 1998). These approaches have been used to increase tumour oxygenation sensitizing to radiotherapy and chemotherapy.

Hyperbaric oxygenation (HBO) increases the oxygen supply to hypoxic tumour cells independent of its blood flow. Thus, HBO has been used clinically in combination with radiotherapy, but the previous combination method in which irradiation was administered during HBO exposure was both hazardous to patients and complex (Dische, 1978; Jain, 1990). As a result, HBO has not been routinely adopted with radiotherapy to treat patients with cancer. We irradiated human malignant gliomas 15 min after HBO exposure based on the hypothesis that elevated partial oxygen tension ($PO_2$) in the tumours was maintained for substantial periods after decompression (Kohshi et al, 1996). Using invasive measurements, it was reported that tissue $PO_2$ increased slowly during HBO exposure and that the decline in $PO_2$ after HBO was slower in subcutaneous tissues than in muscles (Wells et al, 1977), but no study on $PO_2$ change of tumours after HBO has been reported. The purpose of this study was to non-invasively monitor the tumour $PO_2$ changes produced by HBO exposure using MRI, and to clarify whether the elevated oxygen level in the tumours is maintained for substantial periods after decompression.

*Received 24 April 1999*
*Revised 1 July 1999*
*Accepted 8 July 1999*

*Correspondence to:* Y Kinoshita

### MATERIALS AND METHODS

### Phantom preparation

To investigate the effect of oxygen dissolved in water, we measured the proton relaxation time of five phantoms. Small tubes were prepared with different oxygen concentrations. The water phantoms were as follows: (a) water without oxygen, (b) water with bubbling oxygen under 1.0 atmosphere absolute (ATA), (c) water with bubbling oxygen under 1.5 ATA, (d) water with bubbling oxygen under 2.0 ATA, and (e) water with bubbling oxygen under 2.5 ATA. MR spectroscopic measurements were performed using a Spectroscopy Imaging Systems Corporation (SISCO, Varian NMR Instruments, Palo Alto, CA, USA) 4.7 Tesla, 40 cm bore system. The hydrogen-1 resonant frequency was 200.43 MHz. The T1 relaxation time was measured by alteration of the inversion time (TI) using an inversion recovery pulse sequence. An exponential fitting was utilized to calculate T1 relaxation time:

Signal intensity = Mo [1–2 exp (TI/T1)], where Mo = longitudinal magnetization at equilibrium.

### Tumour model

Ten- to 12-week-old female C3H/He mice were used. The research was conducted according to the principles described in the 'Guiding Principles for the Care and Use of Animals approved by the Faculty Meeting of the University of Occupational Environmental Health'. SCCVII tumour cells, the hypoxic fraction of which was 9.1% (Shibamoto et al, 1994), were maintained in culture in RPMI-1640 (Gibco Laboratories, Grand Island, NY, USA) supplemented with 10% fetal bovine serum (Gibco Laboratories) and antibiotics, and trypsinized before making single cell suspensions. The mice were inoculated in the left leg with $3 \times 10^5$ viable SCCVII tumour cells. Urethane-anaesthetized, spontaneously breathing mice were studied when the tumour size was about 1 cm in diameter. During MRI measurements, their legs and tumour were restrained in alginate impression material without occluding the blood supply on the table graduating scale. The mice were transferred with the table into a small experimental hyperbaric chamber. We attempted to set the same position, within 0.5 mm difference between pre- and post-HBO exposure. The temperature was maintained using warm oxygen forced through a hyperbaric chamber during HBO exposure.

### MRI measurements of tumours

MRI measurements were performed using the same MR system described above. MR images were taken with a bird cage-type resonator (inner diameter, 8.9 cm) in a magnet fitted with an actively shielded gradient coil (1.8 G $cm^{-1}$). The molecular oxygen-enhanced MR images were acquired using an inversion recovery-preparation fast low angle shot (IR-FLASH) sequence. The acquisition parameters for the IR-FLASH sequence were as follows: repetition time (TR), 30 ms; echo time (TE), 8 ms; flip angle, 30°; field of view (FOV), 80 × 80 mm; matrix, 128 × 128; one excitation; slice thickness, 2.0 mm. The inversion time was 1000 ms to sensitize the acquisition to the paramagnetic effects of molecular oxygen. Each image of IR-FLASH took about 5 s to acquire. The acquisition parameters for the spin echo (SE) sequence were as follows: FOV, 80 × 80 mm; matrix, 256 × 128; two excitation average; slice thickness, 2.0 mm. For T1-weighted images and gadolinium (Gd)-enhanced images, TR was 300 ms and TE was 20 ms; for the T2-weighted images TR was 2000 ms and TE was 80 ms. The resonance frequency and shimming did not change between the pre- and post-HBO exposure.

### Experimental schedule

For T1-weighted images, a slice was selected through the centre of the tumour and two baseline IR-FLASH images were initially acquired while the mice were breathing air. For the HBO-treated group ($n$ = 6), HBO exposure was given in a small experimental hyperbaric chamber according to the following schedule: 10 min of compression with oxygen, 60 min of 100% oxygen inhalation at 2.0 ATA, and 10 min of decompression with oxygen inhalation. For the normobaric group ($n$ = 5), oxygen inhalation was given in the same schedule as above but without compression. With air inhalation, the acquisition of IR-FLASH images was started 5 min after decompression, and images in both groups were obtained every 2.5 min. Finally, T1- and T2-weighted SE images were obtained. Subsequently, after gadopentetate dimeglumine (Gd-DTPA, 0.4 ml $kg^{-1}$) (Magnevist, Berlex Laboratories, Wayne, NJ, USA) was administered intravenously, the Gd-enhanced image was taken. Tumours with a haemorrhagic lesion on the T1-weighted SE image or necrotic tissue on the T2-weighted SE image were excluded from this study. A region of interest encompassing the tumour image was chosen and the average pixel intensity was calculated.

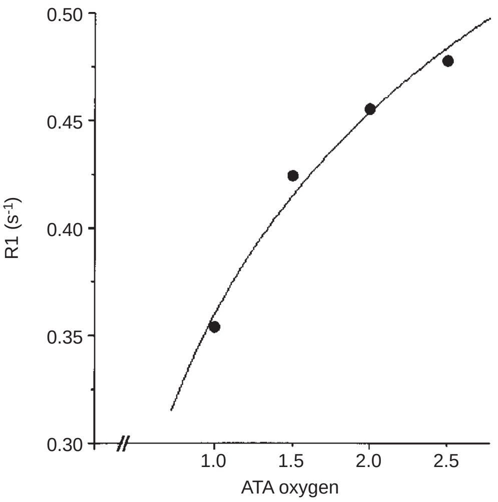

**Figure 1** The relaxation rate (R1 = 1/T1) increased logarithmically with pressure at 25°C. ATA: atmosphere absolute

### RESULTS

### Phantom study

T1 relaxation time of water protons was related to the presence of paramagnetic molecular oxygen dissolved in water. The T1 relaxation time for each water phantom at 25°C was as follows: 3.12 ± 0.06 s without oxygen, 2.83 ± 0.04, 2.36 ± 0.01, 2.20 ± 0.02, 2.09 ± 0.02 s with oxygen under 1.0, 1.5, 2.0 and 2.5 ATA respectively. The relaxation rate (R1 = 1/T1) of pure water without dissolved oxygen was 0.32, and a non-linear relationship ($r^2$ = 0.981) was observed between R1 and ATA (Figure 1). The phantom study indicated that the T1 relaxation time was shortened by dissolved molecular oxygen under the high-pressure environment.

### Tumour study

T1-weighted SE images of pre- and post-treatment of HBO exposure revealed the same registration (Figure 2 A,B), and T2-weighted SE images demonstrated no necrotic lesions (Figure 2C). The Gd-enhanced image showed a homogeneous enhanced tumour of 1 cm in diameter (Figure 2D). The pathological specimen stained haematoxylin and eosin (Figure 2E) showed no evidence of haemorrhage or necrosis. Immediately after HBO exposure, IR-FLASH signals from the tumours of HBO-treated mice showed a signal increase in the tumour compared with the pre-HBO image (Figure 2 F–K). Compared to the two baseline IR-FLASH images, the average signals of

*© 2000 Cancer Research Campaign*

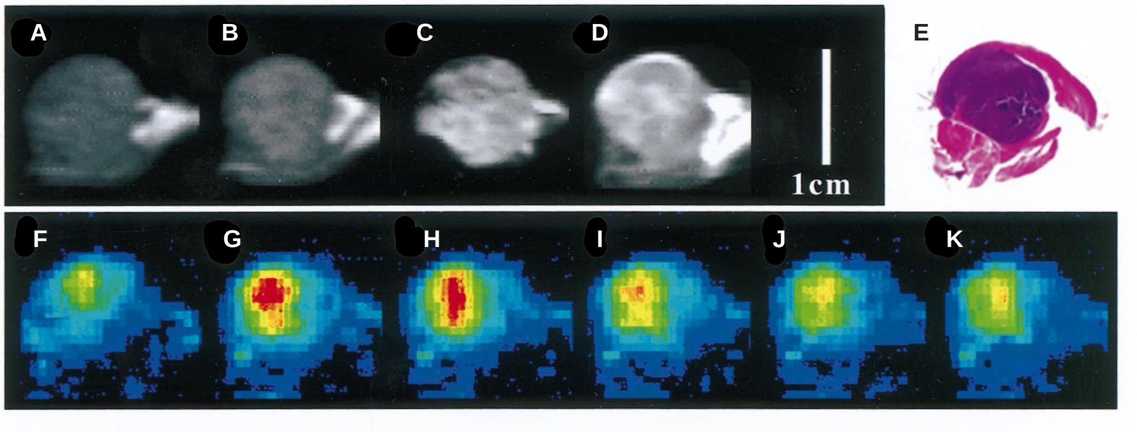

**Figure 2** Demonstrative MR images of tumour-bearing hind leg of HBO-treated (2.0 ATA 100% $O_2$) mice. T1-weighted SE images (**A**: pretreatment, **B**: post-treatment) revealed the same registration and slight signal increase on the image after HBO exposure. T1-weighted SE image (**A, B**), T2-weighted image (**C**), Gd-enhanced image (**D**) and haematoxylin and eosin-stained pathological specimen (**E**) showed no evidence of haemorrhagic or necrotic tissue. Temporal signal changes were demonstrated on IR-FLASH images (**F**: pretreatment, **G**: 5 min after HBO, **H**: 15 min, **I**: 30 min, **J**: 60 min, **K**: 90 min)

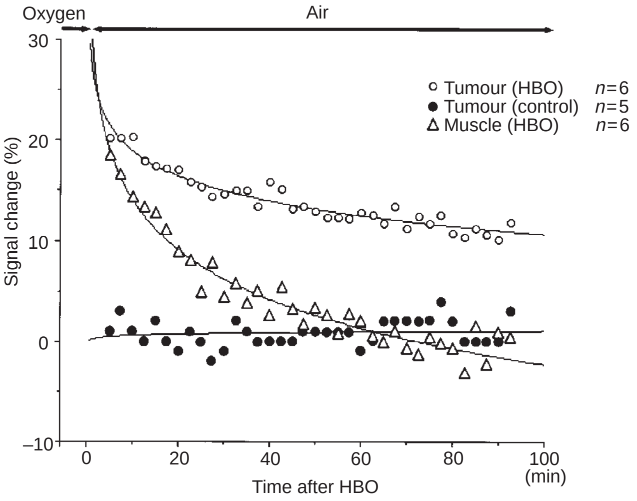

**Figure 3** Temporal signal change of tumours after 2.0 ATA 100% $O_2$ (open circles), muscles after 2.0 ATA 100% $O_2$ (open triangles), and tumours after 1.0 ATA 100% $O_2$ (filled circles). After the HBO treatment, the signals of tumours and muscles were logarithmically decreased, but the tumour signal showed a slower decline than that of the muscle signal. The mean signal elevation of the tumours after 100% 2.0 ATA $O_2$ compression lasted for more than 60 min tumours exposed to HBO showed 20%, 18%, 15%, 13% and 10% increases and those of the muscles demonstrated 18%, 11%, 5%, 0% and –2% in each image intensity at 5, 15, 30, 60 and 90 min after decompression respectively. There was a logarithmic relationship ($r^2$ = 0.929) between the MR signal intensity of tumours and time. Similarly, the average signals from muscles exposed to HBO showed a logarithmic relationship ($r^2$ = 0.946). In contrast, the signals from the tumours in the normobaric group showed no significant change during the course of measurement with air breathing. It is also noteworthy that the MR signal increase of the tumours lasted over 60 min after decompression in the HBO-treatment group, unlike that of the muscle tissues (Figure 3).

### DISCUSSION

Non-invasively, we detected that the decline of MR signal intensity of the tumour was slower than that of muscle using T1-weighted imaging during air-inhalation after HBO decompression. The first study using this non-invasive method examined the effect of hyperoxia on T2*-weighted images of rat R3230AC mammary adenocarcinomas (Karczmar et al, 1994). The same group reported that T2*-weighted images differentiated tumours from normal tissue (Kuperman et al, 1995). They reported that significant signal increases were observed within the tumour centre and rim, while little change was observed in muscle during hyperoxia. Using the same T2*-weighted gradient echo images, another study on the responses of six rodent tumours to carbogen (95% oxygen/ 5% carbon dioxide) suggested that the MR signals were consistent with an increase in oxygen content of blood, tumour cell oxygenation and tumour blood flow (Robinson et al, 1997). On the other hand, using T1-weighted images instead of T2*-weighted images, semi-quantitative measurements of the tumour oxygen level have been discussed (Edelman et al, 1996; Tadamura et al, 1997; Obata et al, 1998). Tadamura (1997) reported that there was no significant change in the T2 value during oxygen inhalation in the tissues, including the spleen and myocardium, in which T1 shortening was observed. These results indicate that T1-weighted imaging is more useful to evaluate the effect of tissue oxygenation compared to T2*-weighted imaging which was affected by blood oxygenation, blood flow and tissue oxygenation.

### Mechanisms affecting MR signal intensity

Two major mechanisms affect MR signal changes in tissue oxygenation. The first mechanism is blood oxygenation level-dependent (BOLD) contrast based on paramagnetic deoxyhaemoglobin and the second is paramagnetic molecular oxygen itself containing two unpaired electrons. Paramagnetic deoxyhaemoglobin in blood creates magnetic susceptibility gradients near blood vessels that produce phase dispersion of water proton magnetization in the surrounding tissue, so the gradient recalled echo-type sequences are very sensitive to the BOLD effect (Ogawa et al, 1990). This BOLD contrast has been utilized to evaluate regional blood flow and/or tissue oxygenation on functional MR imaging. During oxygen inhalation, the mean enhancement on T2*-weighted brain images in the grey matter and the white matter were 4.23% and 1.92% respectively, but T1-weighted

© 2000 Cancer Research Campaign turbo-FLASH images demonstrated no significant changes with a conventional MR scanner at 1.5 Tesla (Berthezéne et al, 1995). This result demonstrated that these local signal increases were attributed to changes in net conversion of deoxyhaemoglobin to oxyhaemoglobin and cerebral blood volume on T2*-weighted images. Although deoxyhaemoglobin is paramagnetic, it does not cause significant T1 shortening. Since the electron spin relaxation time of deoxyhaemoglobin is very short and because water molecules are unable to approach the haem iron within a distance of 3 Å, the T1 of an aqueous solution of deoxyhaemoglobin is not short (Singer and Crooks, 1978). Therefore, T2*-weighted MR images were mainly affected by the BOLD effect of intravascular deoxyhaemoglobin, but T1-weighted MR images were affected by paramagnetic molecular oxygen itself. Moreover, on T1-weighted turbo-FLASH images, no signal change during oxygen inhalation suggested a nearly stable concentration of free oxygen in blood (Berthezéne et al, 1995). Free oxygen in blood represents less than 0.3% and is not significantly modified by oxygen inhalation until the haemoglobin is fully saturated. The molecular oxygen is dissolved into the fluid in proportion to Henry's law and is expressed as $PO_2 \times 0.003$. If $PO_2$ values of the arterial blood before and during inhalation of 100% oxygen are ~100 mmHg and ~500 mmHg, respectively, the concentration of dissolved molecular oxygen will increase by approximately five times. Under 2.5 ATA, the $PO_2$ value of the arterial blood was ~1500 mmHg and the concentration of dissolved oxygen will increase by approximately 15 times (Wells et al, 1977). It was considered that a large quantity of dissolved molecular oxygen shortened the T1 relaxation time of blood in proportion to ATA. Our in vitro study using water phantom demonstrated that it shortened the T1 relaxation time by about 3 s to 2 s according 1–2.5 ATA at 4.7 Tesla magnetic field. Our MR parameters of the IR-FLASH sequence were sensitive to these T1 changes because the image contrast was acquired about 3 s after the inversion pulse.

Our observed T1 changes, however, reflected alterations of dissolved oxygen not only in intravascular blood but also in the fluid of extravascular spaces. We did not measure the intravascular blood $PO_2$ but Wells (1977) demonstrated rapid $PO_2$ decline of the arterial blood and it reached a baseline level within a few minutes after the end of HBO exposure. Since we started the acquisition of IR-FLASH images 5 min after decompression, our observed MR signal changes did not seem to be affected by the arterial blood $PO_2$, but indicated an alternation of extravascular oxygenation. It is noteworthy that oxygen in the extravascular space is important for the effectiveness of radiotherapy.

### Tumour oxygenation method

Many studies to improve tumour oxygenation have been performed. Some investigators have reintroduced the clinical use of carbogen to improve tumour oxygenation. Carbogen breathing may improve tumour blood oxygenation in two ways: (1) the 95% oxygen may simply increase the arterial blood $PO_2$; (2) 5% carbon dioxide may induce vasodilation of afferent tumour vessels (Robinson et al, 1995). Carbogen breathing caused increases of up to 100% in normalized MR image intensity in GH3 prolactinomas grown in rats, and reversion to air breathing caused a subsequent fall in MR image intensity. These changes in signal intensity are consistent with an increase in oxygen content of the blood, tumour cell oxygenation and blood flow of the tumour. Using the Eppendorf $PO_2$ histography, however, normobaric oxygen and carbogen caused no significant change in tumour oxygenation, whereas HBO and hyperbaric carbogen led to improvement of oxygenation (Brizel et al, 1995). Moreover, hyperbaric carbogen was less effective than HBO in increasing the tumour because of the result of adrenergic stimulation from the inspired carbon dioxide. HBO might be the most effective method to reduce tumour hypoxia by increasing the amount of dissolved oxygen in the plasma and tumour cells.

The changes in tissue $PO_2$ reduction after HBO exposure depend on blood flow and/or oxygen consumption in tissues. Wells (1977), using a mass spectrometer probe that quantified the duration and magnitude of the HBO effect, found that tissue $PO_2$ changed slowly during and after HBO exposure and that the decline in $PO_2$ was slower in subcutaneous tissue than in muscle. They concluded that the different $PO_2$ changes in tissues were affected by differences in tissue perfusion. On the other hand, Hall (1994) emphasized poor tissue perfusion in the presence of hypoxic tumour cells (Thomlinson and Gray, 1955). In this study, an increased oxygen level in the tumour continued for a substantial period despite fast oxygen reduction in the muscle. Although the oxygen metabolism in tumours and muscles was not measured in this condition, one of the reasons for the slower $PO_2$ decline in tumours was probably the lower blood flow in tumours.

### Clinical application of HBO

Most malignant tumours appear to have a hypoxic core, and the elimination of this core may destroy cells which are not killed by usual radiation procedures and which may be responsible for recurrence. It is well known that hypoxic tumour cells are resistant to some types of chemotherapy and radiotherapy. Tumour oxygenation is a critical determinant of many forms of cancer therapy. Clinical trials of radiotherapy during HBO showed improvements in the local cure and survival rates of cancers in the head, neck, and uterine cervix and evidence of HBO benefits has been obtained in carcinomas of the bronchus, but not of the bladder (Dische, 1978). Since HBO has an enhancing radiation effect on normal tissues as well as tumours, controversy remains concerning the actual improvement in the therapeutic efficacy of HBO. In addition, the dose correction of radiation absorbed in the chamber wall is complex. But our new protocol that involved irradiation immediately after HBO exposure was simple and safe compared to radiotherapy during HBO exposure (Kohshi et al, 1996). The results of the present non-invasive study supported the theory that elevated MR signal intensity indicates tissue $PO_2$ in the tumours was maintained for substantial periods after decompression. Therefore, irradiation immediately after decompression is considered to be effective for malignant tumours without exerting the influence of radiation on normal tissue. Furthermore, multivariate analysis in our small series revealed that combination with HBO was a good predictive prognostic factor for survival (Kohshi et al, 1999).

### ACKNOWLEDGEMENTS

This work was supported by a grant from Daido Hoxan Inc., Tokyo, Japan, and a Grant-in-Aid for Scientific Research 10877221, from the Ministry of Education, Science and Culture, Japan. We thank Rieko Maeda for preparing the histological sections and Norio Iriguchi for expert technical assistance.

*© 2000 Cancer Research Campaign*

### REFERENCES

Al-Hallaq HA, River JN, Zamora M, Oikawa H and Karczmar GS (1998) Correlation of magnetic resonance and oxygen microelectrode measurements of carbogen-induced changes in tumor oxygenation. *Int J Radiat Oncol Biol Phys* **41**: 151–159

Berthezéne Y, Tournut P, Turjman F, N'Gbesso R, Falise B and Froment JC (1995) Inhaled oxygen: a brain MR contrast agent? *Am J Neuroradiol* **16**: 2010–2012

Brizel DM, Lin S, Johnson JL, Brooks J, Dewhirst MW and Piantadosi CA (1995) The mechanisms by which hyperbaric oxygen and carbogen improve tumour oxygenation. *Br J Cancer* **72**: 1120–1124

Brizel DM, Scully SP, Harrelson JM, Layfield LJ, Dodge RK, Charles HC, Samulski TV, Prosnitz LR and Dewhirst MW (1996) Radiation therapy and hyperthermia improve the oxygenation of human soft tissue sarcomas. *Cancer Res* **56**: 5347–5350

Collingridge DR, Young WK, Vojnovic B, Wardman P, Lynch EM, Hill SA and Chaplin DJ (1997) Measurement of tumor oxygenation: a comparison between polarographic needle electrodes and a time-resolved luminescence-based optical sensor. *Radiat Res* **147**: 329–334

Dische S (1978) Hyperbaric oxygen: the Medical Research Council trials and their clinical significance. *Br J Radiol* **51**: 888–894

Edelman RR, Hatabu H, Tadamura E, Li W and Prasad PV (1996) Noninvasive assessment of regional ventilation in the human lung using oxygen-enhanced magnetic resonance imaging. *Nat Med* **2**: 1236–1239

Hall EJ (1994) *Radiobiology for the Radiologist*, pp. 133–152. Lippincott: Philadelphia

Helmlinger G, Yuan F, Dellian M and Jain RK (1997) Interstitial pH and $PO_2$ gradients in solid tumors in vivo: high-resolution measurements reveal a lack of correlation. *Nat Med* **3**: 177–182

Jain KK (1990) *Texbook of hyperbaric medicine*, pp. 408–417. Hogrefe & Huber Publishers: Toronto

Karczmar GS, River JN, Li J, Vijayakumar S, Goldman Z and Lewis MZ (1994) Effects of hyperoxia on T2* and resonance frequency weighted magnetic resonance images of rodent tumours. *NMR Biomed* **7**: 3–11

Kohshi K, Kinoshita Y, Terashima H, Konda N, Yokota A and Soejima T (1996) Radiotherapy after hyperbaric oxygenation for malignant gliomas: a pilot study. *J Cancer Res Clin Oncol* **122**: 676–678

Kohshi K, Kinoshita Y, Imada H, Kunugita N, Abe H, Terashima H, Tokui N and Uemura S (1999) Effects of radiotherapy after hyperbaric oxygenation on malignant gliomas. *Br J Cancer* **80**: 236–241

Kuperman V, River JN, Lewis MZ, Lubich LM and Karczmar GS (1995) Changes in T2*-weighted images during hyperoxia differentiate tumors from normal tissue. *Magn Reson Med* **33**: 318–325

Obata T, Saito K, Iwasawa T, Hirono K, Yoshida T and Matsubara S (1998) Dynamic MRI of transcorneal dispersion of oxygen into the anterior chamber of human eye. *J Magn Reson Imaging* **8**: 508–510

Ogawa S, Lee TM, Nayak AS and Glynn P (1990) Oxygenation-sensitive contrast in magnetic resonance image of rodent brain at high magnetic fields. *Magn Reson Med* **14**: 68–78

Oikawa H, Al-Hallaq HA, Lewis MZ, River JN, Kovar DA and Karczmar GS (1997) Spectroscopic imaging of the water resonance with short repetition time to study tumor response to hyperoxia. *Magn Reson Med* **38**: 27–32

Rampling R, Cruickshank G, Lewis AD, Fitzsimmons SA and Workman P (1994) Direct measurement of $PO_2$ distribution and bioreductive enzymes in human malignant brain tumours. *Int J Radiat Oncol Biol Phys* **29**: 427–431

Robinson SP, Howe FA and Griffiths JR (1995) Non-invasive monitoring of carbogen-induced changes in tumour blood flow and oxygenation by functional magnetic resonance imaging. *Int J Radiat Oncol Biol Phys* **33**: 855–859

Robinson SP, Rodrigues LM, Ojugo AS, McSheehy PM, Howe FA and Griffiths JR (1997) The response to carbogen breathing in experimental tumour models monitored by gradient-recalled echo magnetic resonance imaging. *Br J Cancer* **75**: 1000–1006

Shibamoto Y, Kitakabu Y, Murata R, Oya N, Shibata T, Sasai K, Takahashi M and Abe M (1994) Reoxygenation in the SCCVII tumor after KU-2285 sensitization plus single or fractionated irradiation. *Int J Radiat Oncol Biol Phys* **29**: 583–586

Singer JR and Crooks LE (1978) Some magnetic studies of normal and leukemic blood. *Clin Eng* **3**: 357–363

Tadamura E, Hatabu H, Li W, Prasad PV and Edelman RR (1997) Effect of oxygen inhalation on relaxation times in various tissues. *J Magn Reson Imaging* **7**: 220–225

Thomlinson RH and Gray LH (1955) The histological structure of some human lung cancers and the possible implications for radiotherapy. *Br J Cancer* **9**: 539–549

Vaupel P, Frinak S and O'Hara M (1984) Direct measurement of reoxygenation in malignant mammary tumors after a single large dose of irradiation. *Adv Exp Med Biol* **180**: 773–782

Wells CH, Goodpasture JE, Horrigan DJ and Hart GB (1977) Tissue gas measurements during hyperbaric oxygen exposure. In: *Proceedings of the 6th International Congress on Hyperbaric Medicine*, Smith (ed), pp. 118–124. Aberdeen University Press: Aberdeen

© 2000 Cancer Research Campaign
